# Dream recall in cognitively healthy older adults: associations with blood *p*‐tau217 levels, *APOE* ε4 carriage, and cognitive decline

**DOI:** 10.1002/alz70857_097922

**Published:** 2025-12-24

**Authors:** Darren M. Lipnicki, Meritxell Valentí, Elizabeth Valeriano‐Lorenzo, Ashleigh S. Vella, Marian Zea‐Sevilla, Mario Ricciardi, Belén Frades, Minerva Martinez, Sonia Wagner, Perminder S. Sachdev, Teodoro del Ser, Pascual Sanchez‐Juan

**Affiliations:** ^1^ Centre for Healthy Brain Ageing (CHeBA), UNSW Sydney, Sydney, NSW, Australia; ^2^ Alzheimer's Center Reina Sofia‐CIEN Foundation, Madrid, Madrid, Spain; ^3^ CIEN Foundation, Reina Sofia Alzheimer Center, ISCIII, Madrid, Madrid, Spain; ^4^ Alzheimer's Center Reina Sofia‐CIEN Foundation, Madrid, Spain

## Abstract

**Background:**

Evidence suggests that dreaming is subserved by the default mode network (DMN) (Domhoff, 2022. *The Neurocognitive Theory of Dreaming*). β‐amyloid plaques and phosphorylated tau (*p*‐tau) protein tangles accumulate and contribute to abnormal functionality in the DMN long before clinical Alzheimer's disease, and similarly early abnormal DMN functionality is shown by apolipoprotein E (*APOE*) gene ε4 allele carriers. Given this, we reasoned that dream recall in cognitively normal older adults may be associated with blood *p*‐tau levels, *APOE* ε4 carriage, and future cognitive decline.

**Method:**

Data were for 1049 cognitively unimpaired individuals (mean age=74.7 years; 64% women) in the Spanish Vallecas Project. Dream recall was ascertained by asking “Do you remember your dreams?” (yes/no). Blood *p*‐tau217 levels >0.247 pg/mL were considered high, and ≥1 ε4 alleles indicated *APOE* ε4 carriage. Cognition was assessed with a modified Preclinical Alzheimer Cognitive Composite (PACCm) annually over 10 years, with z‐scores <‐1 considered relatively low baseline cognition. Our analyses used general linear models, as well as a linear mixed‐effect model to compare the longitudinal cognitive trajectories of dream recallers and non‐recallers. Analyses were controlled for factors including sleep characteristics, medications, depression, and memory test scores.

**Result:**

Thirty‐one percent of participants did not remember dreams. Higher *p*‐tau217 levels and *APOE* ε4 carriage were both associated with a lower likelihood of remembering dreams (OR=0.52, 95%CI=0.35‐0.79, *p* = .002 and OR=0.61, 95%CI=0.43‐0.86, *p* = .006, respectively), independently of memory test scores. Further, individuals who did not remember dreams at baseline showed faster decline in PACCm scores (β_non‐DR_=‐0.03) over 10 years than dream recallers (β_DR_=‐0.02; β_Age*Dream Recall_=0.011, SE=0.005, *p* = .036), despite dream recall status not being associated with relatively low cognition at baseline (OR=0.94, 95%CI=0‐60‐1.48, *p* = .788).

**Conclusion:**

Dream recall status was associated with blood *p*‐tau217 levels, *APOE* ε4 carriage, and future cognitive decline among older individuals cognitively healthy at baseline. Not remembering dreams in later life may be an early indicator of neurodegeneration in the DMN. This would help explain an unexpected and overlooked finding more than 30 years ago that lower dream recall frequency predicted incident dementia (Persson & Skoog, J Geriatr Psychiatry Neurol 1992;5:172‐178), a decade before the DMN was discovered.

